# Analysis of concordance of different haplotype block partitioning algorithms

**DOI:** 10.1186/1471-2105-6-303

**Published:** 2005-12-15

**Authors:** Amit R Indap, Gabor T Marth, Craig A Struble, Peter Tonellato, Michael Olivier

**Affiliations:** 1 Human and Molecular Genetics Center, Medical College of Wisconsin, Milwaukee, USA; 2 Department of Biology, Boston College, Chestnut Hill, USA; 3 Department of Mathematics, Statistics, and Computer Science, Marquette University, Milwaukee, USA

## Abstract

**Background:**

Different classes of haplotype block algorithms exist and the ideal dataset to assess their performance would be to comprehensively re-sequence a large genomic region in a large population. Such data sets are expensive to collect. Alternatively, we performed coalescent simulations to generate haplotypes with a high marker density and compared block partitioning results from diversity based, LD based, and information theoretic algorithms under different values of SNP density and allele frequency.

**Results:**

We simulated 1000 haplotypes using the standard coalescent for three world populations – European, African American, and East Asian – and applied three classes of block partitioning algorithms – diversity based, LD based, and information theoretic. We assessed algorithm differences in number, size, and coverage of blocks inferred under different conditions of SNP density, allele frequency, and sample size.

Each algorithm inferred blocks differing in number, size, and coverage under different density and allele frequency conditions. Different partitions had few if any matching block boundaries. However they still overlapped and a high percentage of total chromosomal region was common to all methods. This percentage was generally higher with a higher density of SNPs and when rarer markers were included.

**Conclusion:**

A gold standard definition of a haplotype block is difficult to achieve, but collecting haplotypes covered with a high density of SNPs, partitioning them with a variety of block algorithms, and identifying regions common to all methods may be the best way to identify genomic regions that harbor SNP variants that cause disease.

## Background

Single Nucleotide Polymorphisms (SNPs) are single base pair differences between individuals in a population. The recent completion of the Human Genome Project has helped facilitate the discovery of millions of SNPs and their use in genetic association studies for human disease [1]. Association studies work on the premise that SNP genotypes are correlated with a disease phenotype. Individual SNPs are genotyped and the frequency of alleles are compared between groups of affected and un-affected individuals. SNPs that are tested for association either must be the causative allele or be in *linkage disequilibrium *(LD) with the causative allele. LD is the non-random association of alleles between adjacent loci [2]. SNPs that are in LD with causative allele serve as a proxy and the association with the disease phenotype is maintained.

Numerous studies have shown that the human genome contains regions of high LD with low haplotype diversity [3-6]. These regions are called *haplotype blocks*. The existence of haplotype blocks reduces the number of SNPs required in association studies by identifying and typing only the subset of tag SNPs which uniquely identify common haplotypes present in a block. The frequencies of these haplotypes can be compared in groups of affected and unaffected individuals [7].

Haplotype blocks are defined computationally by various algorithms and can be classified into three categories: diversity based [3,8], LD-based [6], and information-theoretic [9]. Patil et al. [3] used a diversity based greedy algorithm to partition Chromosome 21 into haplotype blocks in a sample of 20 re-sequenced chromosomes. Their algorithm considers all blocks of consecutive SNPs of one SNP or larger, and defines a haplotype block boundary where at least 80% of observed haplotypes within a block are represented at least one or more times in their sample of chromosomes. Overlapping block boundaries were eliminated by choosing the block with the maximum ratio of SNPs in the block to the number of SNPs required to discriminate all haplotypes represented in the block. The process was repeated until the entire length of the chromosome was partitioned into haplotype blocks. Zhang et al. [8] subsequently provided a dynamic programming implementation for this approach in their software HapBlock [10].

Gabriel et al. [6] used a LD-based algorithm to define haplotype blocks in a worldwide sample of chromosomes from Africa, Asia, and Europe. The authors computed confidence bounds of the value of *D'*, a standard measurement of LD [11], and defined pairs of SNPs to be in strong LD (little evidence of recombination) if the one-sided 95% *D' *confidence bound is between 0.7 and 0.98. The authors defined a haplotype block if least 95% of pairwise SNP comparisons in a region show little evidence of recombination based upon their *D' *confidence bounds. The program Haploview [12] implements this method of Gabriel et al.

Anderson and Novembre [9] use the Minimum Description Length (MDL) principle for defining haplotype blocks which incorporates LD decay between blocks and haplotype diversity within blocks [9]. The MDL principle is an application of information theory to statistical modeling which searches for patterns in data [13]. The description length of a data set is a function of the length with which data can be encoded in binary digits, or bits [9]. The best set of block boundaries defined by Anderson and Novembre's method is the set of block boundaries that has the shortest description length for a set of SNP genotypes that span a genomic region. The authors use a dynamic programming algorithm they call the *iterative dynamic programming algorithm *(IDP) and a faster, but approximate, dynamic programming algorithm called *iterative approximate dynamic programming algorithm *(IADP) to find the minimum description length for a set of haplotypes. Their method is implemented in the program MDBlocks [9].

Previous studies on the empirical performance of block partitioning methods have focused on data sets with differing minor allele frequency cutoffs. The studies of Daly et al. [5], Patil et al. [3], and Gabriel et al. [6] used minor allele frequency cutoffs of 5%, 10%, and 20%, respectfully. Schulze et al. [14] assessed the effects of varying the minor allele frequency cutoff on the number of blocks and tag SNPs inferred by the LD based method of Gabriel et al. [6] and diversity based method of Zhang et al. [8]. As rarer SNPs were removed and the allele frequency cutoff raised, the number of blocks inferred decreased for both methods, showing that the block structure is highly influenced by the allele frequency of SNPs used in their analysis.

Ke et al. [15] studied the impact of SNP density on block boundaries from three different partitioning algorithms: the previously discussed LD approach of Gabriel et al., the four-gamete test [16], and a *D' *threshold approach of Phillips et al [17]. The author's study genotyped over 5000 SNPs in a 10 Mb region of chromosome 20 in four different populations: CEPH families, U.K. Caucasians, African Americans, and East Asians. Block boundaries of the algorithms were assessed with differing marker densities starting at 2 kb and going to 10 kb. Their results show that longer blocks at sparser densities are broken into smaller blocks as more SNPs are added in. Other studies describing the LD block structure of the human genome also used varying marker densities. The study by Phillips et al. [17] on chromosome 19 used an average marker density of one SNP per 17.65 kb with a median value of 5.5 kb. Gabriel et al. [6] used an average density of one SNP every 2 kb. Daly et al. [5] used a density of one marker approximately every 5 kb. Patil et al. [3] used a higher density of SNPs with one SNP every 1.3 kb. This study was also the only one that completely re-sequenced the entire chromosome for all 20 samples.

The ideal data set to fully assess the performance of block partitioning algorithms would be a comprehensively re-sequenced large genomic region in a large number of independent chromosomes. Unfortunately, such data are not available at this time. Only a limited number of samples have been re-sequenced extensively. In addition to the study by Patil et al., as of June 2005 the SeattleSNPs [18] data set has re-sequenced 234 human genes in 24 African-American and 23 European CEPH samples spanning a total of 4868 kb of sequence. The ENCODE project [19] intends to re-sequence five 500 kb genomic regions in the 48 individuals of the HapMap Consortium data set [20].

Therefore, to fully assess the performance of block partitioning algorithms we generated three populations consisting of 1000 haplotypes using the coalescent, a stochastic technique that simulates the genetic history of a sample of chromosomes [11]. Haplotypes representing a 200 kb chromosomal region for three world populations – European, African American, and East Asian – were simulated using an implementation of the coalescent that uses a population-specific demographic history. The population specific profiles we used were previously published in Marth et al. [21], where the authors derive a closed mathematical formula for computing the allele frequency spectrum for a specified demographic profile. The demographic profiles for each of the populations were derived by computing allele frequency spectra predicted by Marth's equation for numerous demographic scenarios and testing the fit between it and the observed spectra from the SNP Consortium data set [1] for each respective population.

In the study presented here, we partitioned our coalescent-derived haplotypes into blocks using the three algorithms described above (diversity based, LD based, information theoretic). We assessed algorithm differences in number, size, and coverage of blocks under different values of marker density, allele frequency, and sample size on the performance of block partitioning algorithms. Our results show a great divergence in haplotype blocks predicted by each method, and supports the notion that it may be advisable to use multiple algorithms in parallel to comprehensively account for all haplotype blocks in the human genome.

## Results

### Data simulation and block partitioning

One thousand haplotypes representing a 200 kb region were generated via the standard coalescent with population specific demographic profiles for three world populations: European, African American, and East Asian. All datasets were analyzed with the three block partitioning algorithms described in the Methods section.

In addition to the complete dataset of 1000 haplotypes, 1000 bootstrap sub-sample replicates of 24 or 96 haplotypes were sampled and filtered for different SNP density (all markers, one marker approximately every 1 kb, one marker approximately every 5 kb) and minor allele frequency (MAF) cutoff values (0.1%, 5%, and 10%). Each bootstrap replicate was partitioned using three methods (HapBlock, Gabriel's method, and MDBlocks). Computer memory constraints prevented MDBlocks from partitioning all 1000 chromosomes using all SNPs for each coalescent-derived population. For the same reason we were only able to analyze 200 bootstrap subsamples of 24 or 96 chromosomes with MDBlocks. More details on coalescent simulations and bootstrap sampling is given in the Methods section of the paper.

### European population partitions using all chromosomes

**Figure 1:**
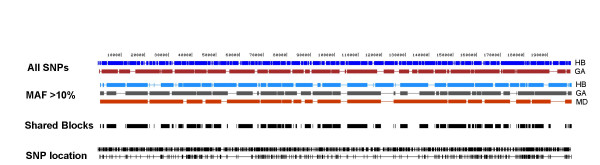
**European population partitions**. European population partitions for all 1000 haplotypes are shown. Block algorithms are abbreviated as HB (HapBlock), GA (Gabriel's method), MD (MDBlocks). The first two tracks show resulting block partitions from HapBlock and Gabriel's method using all SNPs. Next set of three tracks display resulting block partitions using all SNPs with a 10% MAF. The shared blocks track shows chromosomal regions common to all three block partitions using SNPs with a 10% MAF. The last two tracks show SNP positions of all SNPs and SNPs with at least a 10% MAF.

All 1000 European chromosomes were analyzed with HapBlock and Gabriel's method. There were 1349 polymorphic sites with an average SNP density of one SNP per 147 bp. Figure 1 displays the resulting block partitions using all SNPs from the two methods, with the HapBlock partition denoted as HB and Gabriel's method denoted as GA. Table 2 displays descriptive statistics for the HapBlock and Gabriel's method population partitions. No matching block boundaries existed between HapBlock and Gabriel's method. HapBlock inferred a larger number of blocks of smaller physical length than Gabriel's method, but 74% of the sequence was common to blocks inferred by both methods. Both algorithms gave similiar values of coverage, which is defined as sum of the physical haplotype block lengths in base pairs divided by total length of region [22], with values of 85.6% for HapBlock and 86.1% for Gabriel's method, respectively.

**Table 2:** Descriptive block statistics for all 1000 European, African American, and East Asian haplotypes using all SNPs and all SNPs with a MAF of at least 10%

European haplotypes					
Method	MAF	Number of Blocks	Mean bp/block	Mean SNPs/block	% coverage

HapBlock	0.1%	180	951.63	7.49	85.6%
HapBlock	10%	48	3363.60	7.64	80.7%
Gabriel	0.1%	39	4414.28	31.41	86.1%
Gabriel	10%	33	4837.52	10.84	79.8%
MDBlocks	0.1%	-	-	-	-
MDBlocks	10%	18	9348.78	20.39	84.4%
					

African American haplotypes					

Method	MAF	Number of Blocks	Mean bp/block	Mean SNPs/block	% coverage

HapBlock	0.1%	232	733.52	7.13	85%
HapBlock	10%	61	2499.93	6.26	76.2%
Gabriel	0.1%	40	4433.62	37.85	88.6%
Gabriel	10%	36	4416.75	10.25	80%
MDBlocks	0.1%	-	-	-	-
MDBlocks	10%	18	10004.44	21.22	90%
					

East Asian haplotypes					

Method	MAF	Number of Blocks	Mean bp/block	Mean SNPs/block	% coverage

HapBlock	0.1%	208	831.83	7.93	86.5%
HapBlock	10%	57	2596.73	5.84	74%
Gabriel	0.1%	41	3966.02	24.12	81.3%
Gabriel	10%	38	3437.13	8.34	65.3%
MDBlocks	0.1%	-	-	-	-
MDBlocks	10%	18	10006.11	18.50	90%

When analyzing all chromosomes using only SNPs with a MAF of 10% or greater, the total number of markers was reduced to 367 with an average of one SNP every 540 bp. Table 2 also shows descriptive statistics using only SNPs with a MAF of 10% or higher. The number of inferred blocks for HapBlock dropped dramatically from 180 to 48. For Gabriel's method the change was not as large, with 33 blocks inferred. MDBlocks inferred 18 blocks which had the largest physical size. HapBlock, Gabriel's method, and MDBlocks covered 80.7%, 79.8%, and 84.4% of the 200 kb region in blocks. HapBlock again inferred a greater number of blocks of smaller size when compared to the other two methods. Of each possible pair of partitions, only Gabriel's method and MDBlocks contained one set of matching boundaries. Still, a large fraction of sequence, 57%, was common to all three partitions. Table 1 shows percentage of total sequence common to all population block partitions with this population and condition, as well as other populations examined in this study. Figure 1 shows the population partitions for all three methods using only SNPs with at least a MAF 10%, and block regions common for all three algorithms.

**Table 1:** Population partition block overlaps. The table shows the percentage of total sequence common to all three partitions inferred from each algorithm (HapBlock, Gabriel's method, and MDBlocks) for each population studied.

Density	MAF	European	African American	East Asian
all	5%	61%	53%	44%
all	10%	57%	60%	46%
1 kb	0.1%	43%	21%	30%
1 kb	5%	60%	14%	22%
1 kb	10%	53%	17%	26%
5 kb	0.1%	13%	3%	10%
5 kb	5%	29%	0	13%
5 kb	10%	33%	3%	12%

Next, we compared the population partitions of HapBlock and Gabriel's method using all markers vs. all markers with a MAF of at least 10%. For HapBlock, 46% of the blocks inferred with SNPs with the higher MAF were broken up with the addition of rarer markers however, 70.8% of the chromosome is common to both partitions. For Gabriel's method 73% of the sequence is common to partitions resulting from the two differing allele frequency conditions. Only 3% of Gabriel's method blocks were broken into smaller markers with the additon of rarer SNPs.

### African American population partitions using all chromosomes

**Figure 2:**
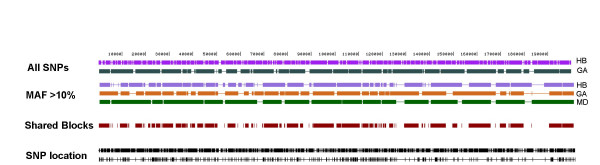
**African American population partitions**. African American population partitions for all 1000 haplotypes are shown. Block algorithms are abbreviated as HB (HapBlock), GA (Gabriel's method), MD (MDBlocks). The first two tracks show resulting block partitions from HapBlock and Gabriel's method using all SNPs. Next set of three tracks display resulting block partitions using all SNPs with a 10% MAF. The shared blocks track shows chromosomal regions common to all three block partitions using SNPs with a 10% MAF. The last two tracks show SNP positions of all SNPs and SNPs with at least a 10% MAF.

A total of of 1653 polymorphic sites with an average density of one SNP every 119 bp defined the 1000 haplotypes in our sample. Table 2 contains descriptive statistics for HapBlock and Gabriel's method partitions using all SNPs. HapBlock identified 232 blocks while Gabriel's method identified 40. Figure 2 displays the HapBlock and Gabriel's method population partitions using all SNPs. Gabriel's method resulted in a slightly larger sequence coverage of 88.6% compared with 85% for HapBlock. HapBlock identified a larger number of blocks of smaller size, however 76%, of the sequence was common to both partitions with no exact matching boundaries between them.

Using only SNPs with a frequency of at least 10% resulted in a total of 382 markers with an average spacing of one SNP every 521 bp. Table 2 also displays descriptive statistics for these block partitions. The number of blocks inferred by HapBlock dropped sharply to 61. For Gabriel's method the difference was smaller with a total of 36 blocks inferred. MDBlocks inferred the smallest number of blocks with 18, but had the largest average size. Percent coverage dropped for HapBlock and Gabriel's method to 76.2% and 80%, respectively. MDBlocks still included 90% of the region in blocks. When comparing all the partitions, 60% of the 200 kb region was common to blocks inferred by all three methods (see Table 1). HapBlock and Gabriel's method shared two matching boundaries, and HapBlock and MDBlocks shared one matching boundary. Figure 2 displays all three block partitions and shared block regions between each partition. Comparing the HapBlock and Gabriel partitions with the full marker set to the corresponding partition of the same method with rarer SNPs filtered out shows that there were common regions identified in both. For HapBlock 67% of the 200 kb region was common to blocks for both conditions. For Gabriel's method 74% of the sequence is included in both partitions.

### East Asian population partitions using all chromosomes

**Figure 3:**
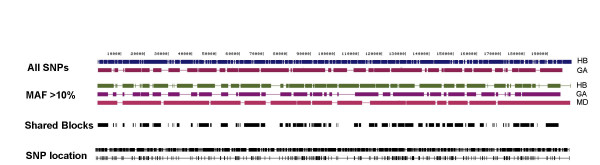
**East Asian population partitions**. East Asian population partitions for all 1000 haplotypes are shown. Block algorithms are abbreviated as HB (HapBlock), GA (Gabriel's method), MD (MDBlocks). First two tracks show resulting block partitions from HapBlock and Gabriel's method using all SNPs. Next set of three tracks display resulting block partitions using all SNPs with a 10% MAF. The shared blocks track shows chromosomal regions common to all three block partitions using SNPs with a 10% MAF. The last two tracks show SNP positions of all SNPs and SNPs with at least a 10% MAF.

A total of 1649 SNPs with an average spacing of one SNP every 120 bp defined the 1000 Asian haplotypes. Table 2 shows descriptive statistics for HapBlock and Gabriel partitions. HapBlock identified 208 blocks, Gabriel's method inferred 41, and 70% of the chromosome was common to block regions inferred by both methods. No matching boundaries existed between the two partitions. Figure 3 shows HapBlock and Gabriel's method block partitions. The HapBlock partition inferred a larger number of blocks of smaller size. Coverage values for HapBlock and Gabriel's method were 86.5% and 81.3%, respectively.

Removing rarer SNPs and using only markers with a MAF of 10% or higher left 333 markers. There was a sharp drop in the number of blocks inferred by HapBlock with 57 blocks compared to 208 when using the full marker set. Gabriel's method inferred 38 blocks. MDBlocks inferred the fewest with 18. None of the partitions shared the same set of SNPs for a block boundary. Table 2 shows descriptive statistics for the resulting block partitions. The amount of sequence coverage drops for two of the methods: 74% for HapBlock and 65.3% for Gabriel's method. Coverage for MDBlocks remains at 90%. The shared block regions between all three methods shown in Figure 3 account for 46% of the chromosomal region.

### Population partitions at other conditions

Descriptive statistics for population partitions of each method at other density and MAF conditions are shown in additional file 7.

### Bootstrap partitions using all markers with frequency of ≥10%

**Figure 4:**
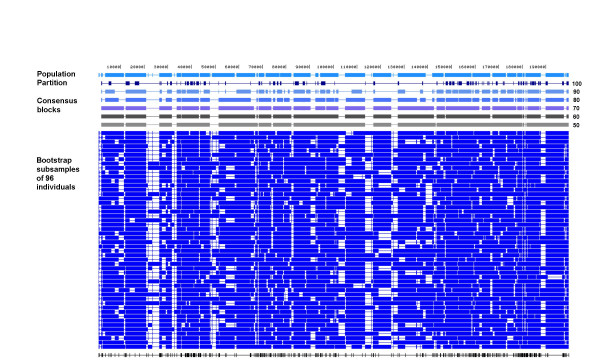
**European HapBlock consensus and bootstrap block partitions**. European HapBlock consensus and bootstrap partitions using all SNPs with at least a 10% MAF are shown. The first track shows the population partition using all 1000 chromosomes followed by consensus blocks defined at thresholds of 100-50% from bootstrap samples of size 96. The next set of tracks are the first 50 individual bootstrap HapBlock partitions.

To assess variation in block structure on more realistic sample sizes (i.e. sample sizes that are being obtained by re-sequencing) we bootstrap subsampled 96 or 24 chromosomes from our original set 1000 times. Figure 4 shows the block partitions resulting from HapBlock for the first 50 individual bootstrap subsamples of size 96 using all SNPs with a MAF of at least 10%. It was clearly evident that the block structure varied between the bootstrap subsamples and the population partition. To find SNPs that were consistently inferred together in blocks above a threshold frequency across all bootstrap subsamples we defined consensus block partitions for HapBlock for threshold values from 100 to 50 percent. (For more details on consensus blocks see Methods.) As the threshold for defining a consensus block is lowered, the physical length of a block increases monotonically and blocks defined at higher thresholds are combined. Table 3 shows the percentage of chromosomal region common to both the population partition and consensus block partitions of HapBlock using only SNPs with a MAF of at least 10%.

**Table 3:** European population and consensus block overlap. Percentage of total sequence common to each method's consensus blocks defined from bootstrap subsamples of 96 chromosomes and population partition using all SNPs with at least a 10% MAF.

consensus threshold	HapBlock	Gabriel's Method	MDBlocks
100	20%	17%	31%
90	55%	45%	66%
80	72%	53%	76%
70	77%	57%	78%
60	78%	61%	81%
50	79%	65%	81%

**Figure 5:**
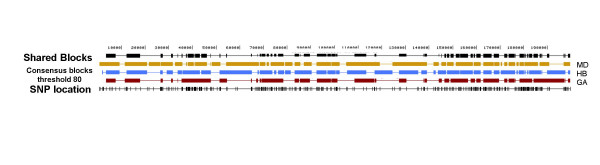
**Common European consensus block regions**. Overlapping consensus block regions from each consensus block defined from MDBlocks (MD), HapBlock (HB), and Gabriel's method (GA). Consensus blocks shown from each method are defined at a threshold of 80% using all SNPs with at least a 10% MAF. The SNP positions are shown in the last track.

Gabriel's method and MDBlocks partitions also showed within population variation in block structure. (See additional files 1 and 2.) Table 3 also contains the percentage of total sequence common between the population partitions of Gabriel's method and MDBlocks, and each consensus block definition. Similar to the HapBlock results as the threshold for defining a consensus block is lowered, the amount block regions common to both partitions increased. Of the three methods, MDBlocks consensus blocks had the greatest amount of total sequence in common with the population partition. Table 4 shows the percentage total sequence common to all three consensus block definitions at each threshold value. Figure 5 displays consensus blocks from each algorithm defined at a 80% threshold, and block regions common to all three consensus blocks. While these common block regions cover only 40% of the 200 kb region in blocks, it was encouraging to find that our consenus block partitions overlapped.

**Table 4:** Consensus block overlap. Percentage of total sequence common to all three European consensus blocks defined from bootstrap subsamples of 96 European haplotypes using all SNPs with at least 10% MAF.

consensus threshold	% common sequence
100	9%
90	30%
80	40%
70	45%
60	51%
50	57%

**Figure 6:**
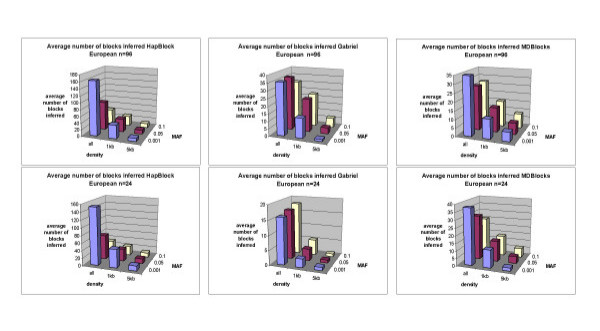
**Average number of blocks inferred European bootstrap subsamples**. Figure 6 shows 3-d bar plots of the average number of blocks inferred for HapBlock, Gabriel's method, and MDBlocks partitions on European bootstrap replicates of sizes 96 and 24 at each SNP density and MAF condition.

**Figure 7:**
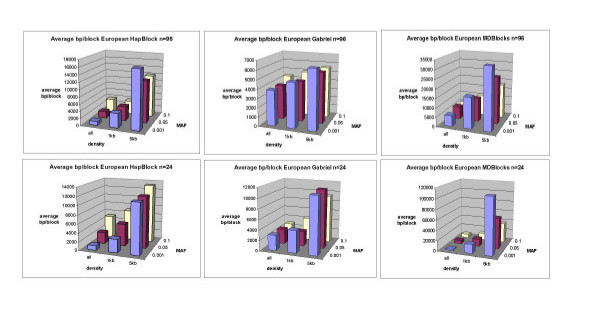
**Average bp/block European bootstrap subsamples**. Figure 7 shows 3-d bar plots of the average number of base pairs per block inferred for HapBlock, Gabriel's method, and MDBlocks partitions on European bootstrap replicates of sizes 96 and 24 at each SNP density and MAF condition.

Figures 6 and 7 show the average number of blocks and base pairs per blocks of each partitioning algorithm tested for European haplotype bootstrap subsample sizes of 24 and 96 chromosomes for other density and MAF conditions. There is an inverse relationship between the number of blocks inferred and their average size in base pairs per blocks as SNP density increased. Coverage generally increased with an increased density of SNPs (see supplementary Figure 3).

Similar patterns for African American and East Asian bootstraps were found. Variation in block structure between bootstrap samples existed. The same pattern of an inverse relationship between the number of blocks and their average size as SNP density increased, remained. As the threshold for a consensus block is lowered, the percentage of sequence common between the population block partition increased monotonically. Also as the threshold is lowered, there is a greater percentage of total sequence common to all consensus blocks defined from each method. (Data not shown).

## Discussion

We generated three populations of haplotypes via coalescent simulations to assess the performance of three block partitioning algorithms under different marker density and allele frequency conditions. Each of the block algorithms employed in this study partitions a genomic region into haplotype blocks using vastly different approaches. In addition to the three algorithms described here, there are other definitions for haplotype blocks not examined [16,17]. Despite all these algorithms, there is no widely accepted definition of how to best define haplotype blocks [23].

The descriptive statistics of each population block partition using all 1000 chromosomes clearly show that results are different in number, size, and coverage of inferred blocks, particularly with a higher density of markers. HapBlock generally inferred the largest number of blocks of smallest size and MDBlocks inferred the fewest number of blocks of largest size. While there are few exact matching block boundaries between different partitions, there is a large amount of common block regions between them. Increasing the density of markers had a more dramatic effect on the percent coverage for Gabriel's method than the other two methods due to fact that LD patterns are sensitive to marker density and can change with the addition of more markers [15]. The amount of coverage, in turn, influences the percentage of total sequence common to all partitions since there is a greater chance of overlap between them.

To assess within population variation in block structure we bootstrap subsampled haplotypes of sizes 24 or 96 chromosomes. The descriptive statistics of the bootstrap partitions indicate that the number of inferred blocks increases as a higher density of markers is used. Also, the average number of base pairs per blocks decreases with a higher density of markers, hence there is an inverse relationship between the number of blocks inferred and their physical size. Removing rarer SNPs does not necessarily decrease the number of blocks inferred for each of the methods when conditioning on a density value. This result is in contrast to the results of Schulze et al. [14], who found that removing rarer SNPs decreased the number of blocks inferred by the HapBlock and Gabriel's method. This maybe a result of the stochastic nature of the coalescent. To get a clearer picture of the effect of allele frequency on the performance of block partitioning algorithms, it may require the simulation of many more genealogies.

Our consensus block definitions attempt to identify SNPs consistently inferred together in blocks across all bootstrap replicates. The amount of common block regions between the population partition and consensus block definitions from bootstrap samples depends heavily on the threshold to define a consensus block, as well as the percent coverage of the bootstrap and population partitions. If a significant proportion of the chromosome is inferred in blocks in both the population and consensus definitions, there is a greater chance of finding common block regions. However as discussed earlier, this attribute is influenced by SNP density and allele frequency of markers.

For Gabriel's method, the number, size, and coverage of inferred blocks varied dramatically between the bootstrap samples and population partitions. The average number of blocks inferred from Gabriel's method for bootstrap samples of size 24 and 96 of European haplotypes, using all markers with a MAF of at least 10% was 18.06 and 30.76, respectively. On average 20.1% and 68.1% of the 200 kb region were inferred in blocks. These numbers differ from the population partition numbers of 33 blocks and 79.8%. These disparate numbers illustrate the effect sample size has on estimating confidence bounds of *D'*. This also explains the fact that in certain bootstrap samples, Gabriel's method failed to infer any blocks. The percent overlap between the consensus blocks defined from bootstrap samples of size 24 and the population partition never exceed 16%, even at the most liberal consensus threshold of 50%. For consensus blocks defined from bootstrap sample sizes of 96, the percent intersection with the population partition is 53% even at the fairly high threshold of 80%. For HapBlock and MDBlocks, differences between average coverage values from the bootstrap experiments to the population partition are not as large, and they showed a larger percentage of sequence intersection between the consensus blocks.

Comparing the consensus blocks for one method to the population partition of the same method addresses the block structure variation within a particular algorithm. To find common block regions in bootstrap subsamples from differing algorithms, we found the overlapping boundaries between consensus block regions from each algorithm. For certain density, MAF conditions, and consensus block thresholds, there was very low or non-existent overlap. These numbers can be severely reduced if a particular method fails to infer a large number of blocks covering a significant portion of sequence, as was the case for Gabriel's method at the sparsest marker density of 5 kb. When using all SNPs with a 10% MAF for European haplotypes, the percentage overlap between all consensus blocks ranges from 9–57% depending on the consensus threshold. Finding block regions common to all three methods is an encouraging sign because each algorithm takes a different approach to the block partitioning problem. If the haplotype block paradigm is an accurate description of underlying LD patterns of the human genome, different algorithms should find common block regions since the three methods base their algorithms on various attributes of the paradigm.

Rather than searching for exact matching boundaries using Schwartz concordance test statistic as a measure of block concordance [24], we chose to compute the percentage of common block regions between two different block partitions as our metric of concordance. While using the block concordance test statistic is a valid approach, the method cannot assess the significance of block boundaries which may differ by few SNPs, but still have a significant degree of overlap between block regions. There was only one matching boundary between each possible of pair of partitions using all 1000 European haplotypes using all SNPs with a MAF of 10%. However, 57% of the 200 kb region was common to blocks defined from all three methods.

In our analysis we focused on the number, size, and coverage of haplotype blocks inferred by three different algorithms. We do not discuss tag SNPs identification because we view it as a separate problem. However, it should be pointed out that the dynamic programming approach of HapBlock is closely tied to tag SNPs because it defines blocks which minimize the number of SNPs needed to distinguish common haplotypes within a block. Recently, a method formulated by Halldorsson et al. [25] selects tag SNPs which does not require a haplotype block definition. Also, the tag SNP algorithm LDselect [26] chooses tag SNPs independent of chosen haplotype block boundaries.

Another point to address in our study design is that the simulated haplotypes used were derived from a single realization of a coalescent simulation, hence our study does not address genetic sampling [27]. Since we bootstrap subsampled 96 or 24 individuals from a population of 1000, we fix the genetic history of our data set and focus on the statistical sampling on the performance of block partitioning algorithms used in this study. We also chose not to vary recombination rate or incorporate recombination hotspots in our simulations since we only analyzed a 200 kb region. Due to these limitations, we did not compare populations to each other. Rather, we examined the trends seen in each population and used coalescent simulations with three different population histories to ensure that the results from the three block partitioning algorithms were not due to the coalescent parameters chosen. The recent study of Ding et al. [28] address the affects of population genetic parameters, such as the mutation and recombination rate, on the diversity and LD based algorithms discussed here for multiple realizations of coalescent genealogies.

## Conclusion

In summary, our results show that for the population partitions using all 1000 chromosomes, there is a varied range of number, size, and coverage of blocks between the different methods. The percentage total sequence common to all three partitioning algorithms ranges from 3–61% depending on the population and is generally higher using a high density of SNPs with a wide range of MAF. Bootstrap sampling of haplotypes from the population shows there is within population variation in block structure for all three methods. Our consensus block definition attempts to define blocks based on sets of SNPs consistently found together in blocks across all bootstrap replicates. Using a higher density of markers there is an increased percentage of total sequence in common with consensus blocks and population partitions. The percentage of common block regions between consensus blocks defined from all three methods is influenced by the percent coverage of individual partitions, which itself is influenced by the density and allele frequency of markers that comprise the haplotypes to be partitioned. It is evident that each algorithm gave a different picture of haplotype block structure at differing density and MAF values and few, if any exactly matching block boundaries existed. An open question that remains is how best to merge or integrate block definitions from different algorithms. For empirical studies, it is advisable to subject collected data to a variety of block algorithms and identify common block regions. If distinct partitioning algorithms show a large portion of overlap in inferred block regions, then these genomic regions can be investigated further to identify genetic variants causing disease.

## Methods

### Coalescent simulations

Haplotypes representing a 200 kb chromosomal region for three world populations, European, African American, and East Asian were generated using an implementation of the standard coalescent with uniform recombination, which uses a population-specific demographic history. The demographic profiles for each of the three populations considered in this study were as determined by Marth et al. from The SNP Consortium genotype data [21]. These profiles are characterized by 3 effective population size epochs. For example, for the European population, we used an ancestral effective population size of 10,000 individuals, followed by a bottleneck phase of an effective population size of 2,000 lasting 500 generations, then an expansion to an effective population size of 20,000 starting 3,000 generations ago. The average number of mutations occurring along a branch of the genealogy (lineage) is Poisson distributed and proportional to the branch length. The value of the (constant) mutation rate *μ *in the simulations was 2.5 × 10-8. Since the effective population size is different within each of the three epochs of the demographic profiles, there is not a single value of *θ*, the scaled mutation rate. The equivalent values of *θ *that take into account the fluctuating effective population size are, for the European population: 9.85 × 10-4; for African American and East Asian populations: 1.29 × 10-3 and 1.03 × 10-3, respectively. For each population, the haplotypes we used to examine the partitioning algorithms were drawn from a single realization of the coalescent.

The coalescent simulation software was implemented in Perl and run on a Sun Blade 1000 with dual 750 MHz Ultra Sparc III processors and 4.5 GB of RAM. To validate the correctness of the program 200 genealogies of 41 individuals for a 200 kb region were simulated. The average frequency spectrum was tabulated from these 200 simulations and plotted against the predicted spectra from Marth's mathematical formula. The results of the observed and predicted spectra for each population is shown in supplementary figures 4, 5, and 6.

### Haplotype block partitioning algorithms

Three categories of block partitioning algorithms were used in the study: diversity based, LD based, and information theoretic. The software programs that implement each method are described below. All three programs were run on a Sun Blade 1000 with dual 750 MHz Ultra Sparc III processors and 4.5 GB of RAM.

#### HapBlock

HapBlock v2.1 is a diversity based algorithm that minimizes the number SNPs that distinguish at least *α *percent of common haplotypes [8]. A haplotype block comprised of at least one SNP is defined if the number of common haplotypes represents at least *α *percent of all the observed haplotypes. A haplotype can be designated common either by its frequency or the number of times represented in the set of observed haplotypes. We chose to designate a haplotype as common if it had a frequency of at least 10%. Hence, for our study we set *α *and *β *to 0.80 and 0.10, respectively. The program is available for download here: .

#### Gabriel's Method

Gabriel's method [6] is implemented in the software Haploview v3.11 [12]. Gabriel's method defines pairs of SNPs to be in strong LD if the one-sided 95% *D' *confidence bound is between 0.7 and 0.98. The method defines a block if 95% of pairwise SNP comparisons are in strong LD. For our study Haploview was executed in command line mode to obtain partitions from Gabriel's method. Executing Gabriel's method on certain bootstrap samples generated a software error. Corresponding with the author for Haploview, we were not able to identify the cause of the error (Jeffery Barrett personal communication). But for all bootstrap samples of haplotypes analyzed, this error was encountered on less than 1% of the time. Haploview is available for download here: .

#### MDBlocks

MDBlocks vl.0 uses the Minimum Description Length (MDL) principle for defining blocks [9]. It considers the set of all possible block boundaries and finds the one with the minimum description length using two versions of a dynamic programming algorithm. The first is called the *iterative dynamic programming algorithm *(IDP) The second is a faster, but approximate method called the *iterative approximate dynamic programming algorithm *(IADP). Due to the number and size of haplotypes analyzed, we used the IADP option. MDBlocks ran out of computer memory when attempting to partition all 1000 haplotypes using all SNPs for each population when using the IADP algorithm. MDBlocks is available for download here: .

### Bootstrap subsampling

To assess variation in the number and size of blocks inferred by the three partitioning algorithms used in the study under differing values of sample size, SNP density, and MAF cutoffs, 1000 bootstrap subsamples of sizes 24 or 96 were drawn with replacement from the population. A true bootstrap sample is one that is the same size as the original sample (1000). Since we are making smaller samples of 96 or 24 chromosomes, it is more properly called a bootstrap subsample. Initially each bootstrap subsample contained the full set of SNPs, and was progressively filtered for each possible pair of SNP density and MAF cutoff values.

Hence, the same set of individuals that make up a particular bootstrap subsample can be compared at differing density and MAF conditions. If a subsample contained a monomorphic site, it was removed prior to the initial filtering of density and allele frequency conditions. Since monomorphic sites do not contain any information, in an information theoretic sense, and information theory forms the basis for MDBlocks, the program would crash. Removing monomorphic SNPs in our bootstrapping routine solved this problem (Eric C. Anderson, personal communication).

### Consensus block definition

To identify SNPs that are consistently inferred together in the same block across all bootstrap subsamples, we introduce the idea of a *consensus block*. Let the collection *P *= *p*_1_, ..., *p*_1000 _be the collection of bootstrap partitions resulting from a particular method. Let *S *be the set of SNPs that comprise the haplotypes. For each *SNP*_*i *_and *SNP*_*i*+1_, we calculate how often they are assigned to the same block across all bootstrap samples. We call this the neighbor probability. We define a *consensus block *as collection of consecutive SNPs whose neighbor probability is greater than or equal to some threshold percentage *t*, for *t *= 100 90 80 70 60 50. Consensus blocks were defined for each of the density and MAF conditions for bootstrap subsamples of sizes 24 and 96. As described in the previous section, if a bootstrap subsample initially contained a monomorphic site, it was removed. However, this leads to the situation that not all bootstrap replicates may contain the same SNPs. To calculate consensus blocks, then we take the union of all markers used across all bootstrap replicates and then proceed to calculate neighbor probability. If a particular SNP was not used in a particular bootstrap, and is a member of a the union set of SNPs, its block assignment was treated as missing data and imputed in the following way. If the adjacent markers to the left and to the right of the missing marker were assigned to the same block number, then the missing SNP in question was assigned to the same block.

### Data storage

All data regarding coalescent-derived haplotypes (SNP positions, allele frequencies, etc), block partitions (number of blocks inferred, block boundaries, etc), and consensus block definitions were stored in tables in a MySQL v3.23 database.

### Visualization of block partitions

Block partitions were visualized in the UCSC Genome Browser [29].

### Block partition intersection

Finding the common regions between different block partitions was achieved by executing the appropriate MySQL query on tables holding information for block partition boundaries. For a subset of density and MAF conditions (all SNPs, all SNPs with a 10% MAF) correctness of the database query was verified by using sequence intersection feature of the UCSC Table Browser [30].

## Authors' contributions

MO conceived the original experimental question. GTM provided the source code for coalescent simulations. ARI, PT, and MO formulated the idea of consensus blocks. CAS offered helpful advice on data analysis and implementation. ARI executed the block partitioning programs, collected and analyzed the data, and wrote the paper with editorial comments and modifications from MO, GTM, CAS, and PT.

## Supplementary Material

Additional File 7 Supplementary file 7 is an Excel sheet containing descriptive statistics for population partitions using all 1000 haplotypes for European, African American, East Asian populations for all SNP density and MAF conditions. Click here for file

Additional File 1 Supplementary Figure 1 shows Gabriel's method consensus and bootstrap partitions using all SNPs with at least a 10% MAF for European haplotypes. The first track shows the population partition using all 1000 chromosomes followed by consensus blocks defined at thresholds of 100-50% from bootstrap samples of size 96. The next set of tracks are the first 50 individual bootstrap Gabriel's method partitions. Click here for file

Additional File 2 Supplementary Figure 2 shows MDBlocks consensus and bootstrap partitions using all SNPs with at least a 10% MAF for European haplotypes. The first track shows the population partition using all 1000 chromosomes followed by consensus blocks defined at thresholds of 100-50% from bootstrap samples of size 96. The next set of tracks are the first 50 individual bootstrap MDBlocks partitions. Click here for file

Additional File 3 Supplementary Figure 3 shows 3-d bar plots of the average coverage of HapBlock, Gabriel's method, and MDBlocks partitions on European bootstrap replicates of sizes 96 and 24 at each SNP density and MAF condition. Click here for file

Additional File 4 Supplementary Figure 4 shows the validated European allele frequency spectrum (AFS). The average folded AFS from 200 coalescent genealogies of 41 individuals is plotted in green. The predicted AFS from Marth's mathematical formula is shown in red. Click here for file

Additional File 5 Supplementary figure 5 shows the validated African American allele frequency specturm (AFS). The average folded AFS from 200 coalescent genealogies of 41 individuals is plotted in green. The predicted AFS from Marth's mathematical formula is shown in red. Click here for file

Additional File 6 Supplementary figure 6 shows the validated East Asian allele frequency specturm (AFS). The average folded AFS from 200 coalescent genealogies of 41 individuals is plotted in green. The predicted AFS from Marth's mathematical formula is shown in red. Click here for file

## References

[B1] Sachidanandam R, Weissman D, Schmidt SC, Kakol JM, Stein LD, Marth G, Sherry S (2001). A map of genome sequence information containing 1.42 million single nucleotide polymorphisms. Nature.

[B2] Weiss KM, Clark AG (2002). Linkage disequilibrium and mapping of complex human traits. Trends in Genetics.

[B3] Patil N, Berno AJ, Hinds DA, Barrett WA, Doshi JM, Hacker CR, Kautzer CR, Lee DH, Marjoribanks C, McDonough DP, Nguyen BTN, Norris MC, Sheehan JB, Shen N, Stern D, Stokowski RP, Thomas DJ, Trulson MO, Vyas KR, Frazer KA, Fodor SPA, Cox DR (2003). Blocks of Limited Haplotype Diversity Revealed by High-Resolution Scanning of Human Chromosome 21. Science.

[B4] Olivier M, Bustos VI, Levy MR, Smick GA, Moreno I, Bushard JM, Almendras AA, Sheppard K, Zierten DL, Aggarwal A, Carlson CS, Foster BD, Vo N, Kelly L, Liu X, Cox DR (2001). Complex High-Resolution Linkage Disequilibrium and Haplotype Patterns of Single-Nucleotide Polymorphisms in 2.5 Mb of Sequence on Human Chromosome 21. Genomics.

[B5] Daly MJ, Rioux JD, Schaffner SF, Hudson TJ, Lander ES (2001). High-resolution haplotype structure in the human genome. Nature Genetics.

[B6] Gabriel SB, Schaffner SF, Nguyen H, Moore JM, Roy J, Blumenstiel B, Higgins J, DeFelice M, Lochner A, Faggart M, Liu-Cordero SN, Rotimi C, Adeyemo A, Cooper R, Ward R, Lander ES, Daly MJ, Altshuler D (2003). The Structure of Haplotype Blocks in the Human Genome. Science.

[B7] Hirschorn JN, Daly MJ (2005). Genome-wide Association Studies for Common Diseases and Complex Traits. Nature Reviews Genetics.

[B8] Zhang K, Deng M, Chen T, Waterman MS, Sun F (2002). A dynamic programming algorithm for haplotype block partitioning. PNAS.

[B9] Anderson EC, Novembre J (2003). Finding Haplotype Block Boundaries by Using the Minimum-Description-Length Principle. American Journal of Human Genetics.

[B10] Zhang K, Qin Z, Chen T, Liu JS, Waterman MS, Sun F (2005). HapBlock: haplotype block partitioning and tag SNP selection software using a set of dynamic programming algorithms. Bioinformatics.

[B11] Nordborg M, Tavare S (2002). Linkage disequilibrium: What history has to tell us. Trends in Genetics.

[B12] Barrett JC, Fry B, Mailer J, Daly MJ (2005). Haploview: analysis and visualization of LD and haplotype maps. Bioinformatics.

[B13] Hansen MH, Yu B (2001). Model Selection and the Principle of Minimum Description Length. Journal of the American Statistical Association.

[B14] Schulze TG, Zhang K, Chen YS, Akula N, Sun F, McMahon FJ (2004). Defining haplotype blocks and tag single-nucleotide polymorphisms in the human genome. Human Molecular Genetics.

[B15] Ke X, Hunt S, Tapper W, Lawrence R, Stavrides G, Ghori J, Whittaker P, Collins A, Morris AP, Bentley D, Cardon LR, Deloukas P (2004). The impact of SNP density on fine-scale patterns of linkage disequilibrium. Human Molecular Genetics.

[B16] Wang N, Akey JM, Zhang K, Chakraborty R, Jin L (2002). Distribution of Recombination Crossovers and the Origin of Haplotype Blocks: The Interplay of Population History, Recombination, and Mutation. American Journal of Human Genetics.

[B17] Phillips M, Lawrence R, Sachidanandam R, Morris A, Balding D, Donaldson M, Studebaker J, Ankener W, Alfisi S, Kuo FS, Camisa A, Pazorov V, Scott K, Carey B, Faith J, Katari G, Bhatti H, Cyr J, Derohannessian V, Elosua C, Forman A, Grecco N, Hock C, Kuebler J, Lathrop J, Mockler M, Nachtman E, Restine S, Varde S, Hozza M, Gelfand C, Broxholme J, Abecasis G, Boyce-Jacino M, Cardon L (2003). Chromosome-wide distribution of haplotype blocks and the role of recombination hot spots. Nature Genetics.

[B18] Carlson CS, Eberle MA, Rieder MJ, Smith JD, Kruglyak L, Nickerson DA (2003). Additional SNPs and linkage-disequilibrium analyses are necessary for whole-genome association studies in humans. Nature Genetics.

[B19] Consortium TEP (2003). The ENCODE (ENCyclopedia Of DNA Elements) Project. Science.

[B20] International HapMap Consortium T (2003). The International HapMap Project. Nature.

[B21] Marth G, Schuler G, Yeh R, Davenport R, Agarwala R, Church D, Wheelan S, Baker J, Ward M, Kholodov M, Phan L, Czabarka E, Murvai J, Cutler D, Wooding S, Rogers A, Chakravarti A, Harpending HC, Kwok PY, Sherry ST (2003). Sequence variations in the public genome data reflect a bottlenecked population history. PNAS.

[B22] Pritchard JK, Wall J (2003). Assessing the Performance of the Haplotype Block Model of Linkage Disequilibrium. American Journal of Human Genetics.

[B23] Bafna V, Halldorsson BV, Schwartz R, Clark AG, Istrail S (2003). Haplotypes and Informative SNP Selection Algorithms: Don't Block Out Information. RECOMB.

[B24] Schwartz R, Halldorsson BV, Bafna V, Clark AG, Istrail S (2003). Robustness of Inference of Haplotype Block Structure. Journal of Computational Biology.

[B25] Halldorsson BV, Bafna V, Lippert R, Schwartz R, Vega FMDL, Clark AG, Istrail S (2004). Optimal Haplotype Block-Free Selection of Tagging SNPs for Genome-Wide Association Studies. Genome Research.

[B26] Carlson CS, Eberle MA, Rieder MJ, Yi Q, Kruglyak L, Nickerson DA (2004). Selecting a Maximally Informative Set of Single-Nucleotide Polymorphisms for Association Analyses Using Linkage Disequilibrium. The American Journal of Human Genetics.

[B27] Weir BS (1996). Genetic Data Analysis II.

[B28] Ding K, Zhou K, Zhang J, Knight J, Zhang X, Shen Y (2005). The Effect of Haplotype-Block Definitions on Inference of Haplotype-Block Structure and htSNPs Selection. Molecular Biology and Evolution.

[B29] Kent WJ, Sugnet CW, Furey TS, Roskin KM, Pringle TH, Zahler AM, Haussler D (2002). The Human Genome Browser at UCSC. Genome Research.

[B30] Karolchik D, Hinrichs AS, Furey TS, Roskin KM, Sugnet CW, Haussler D, Kent WJ (2004). The UCSC Table Browser data retrieval tool. Nucleic Acids Research.

