# Supplementary figures and images for: Analysis of concordance of different haplotype block partitioning algorithms
Source: BMC Bioinformatics. 2005 Dec 15;6:303. doi: 10.1186/1471-2105-6-303 (PMC1343594; doi:10.1186/1471-2105-6-303)

## Slide 1
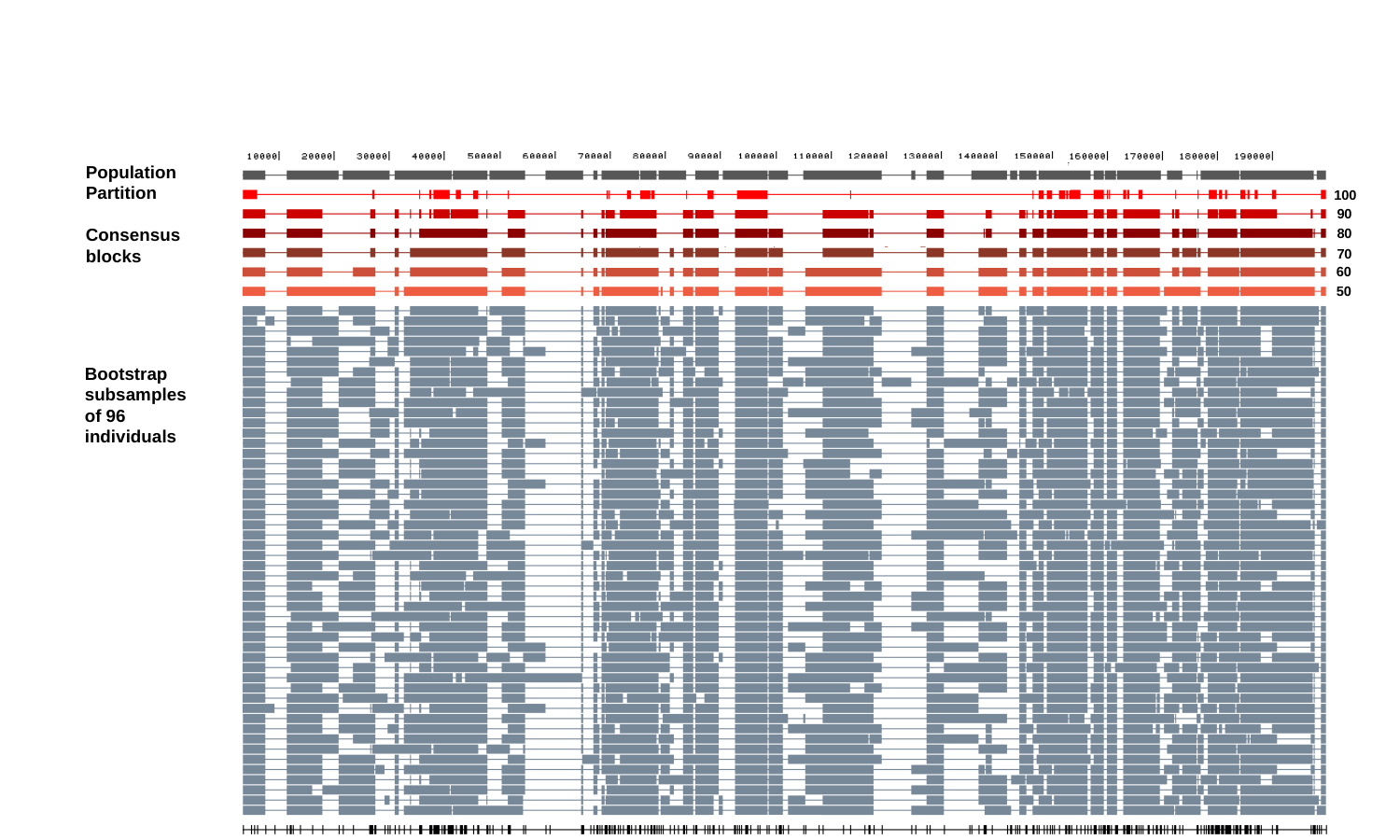

Population Partition
100
90
Consensus blocks
80
70
60
50
Bootstrap subsamples of 96 individuals

Supplement: Additional File 1 — Supplementary Figure 1 shows Gabriel's method consensus and bootstrap partitions using all SNPs with at least a 10% MAF for European haplotypes. The first track shows the population partition using all 1000 chromosomes followed by consensus blocks defined at thresholds of 100-50% from bootstrap samples of size 96. The next set of tracks are the first 50 individual bootstrap Gabriel's method partitions. [file 1471-2105-6-303-S1.ppt]

## Slide 1
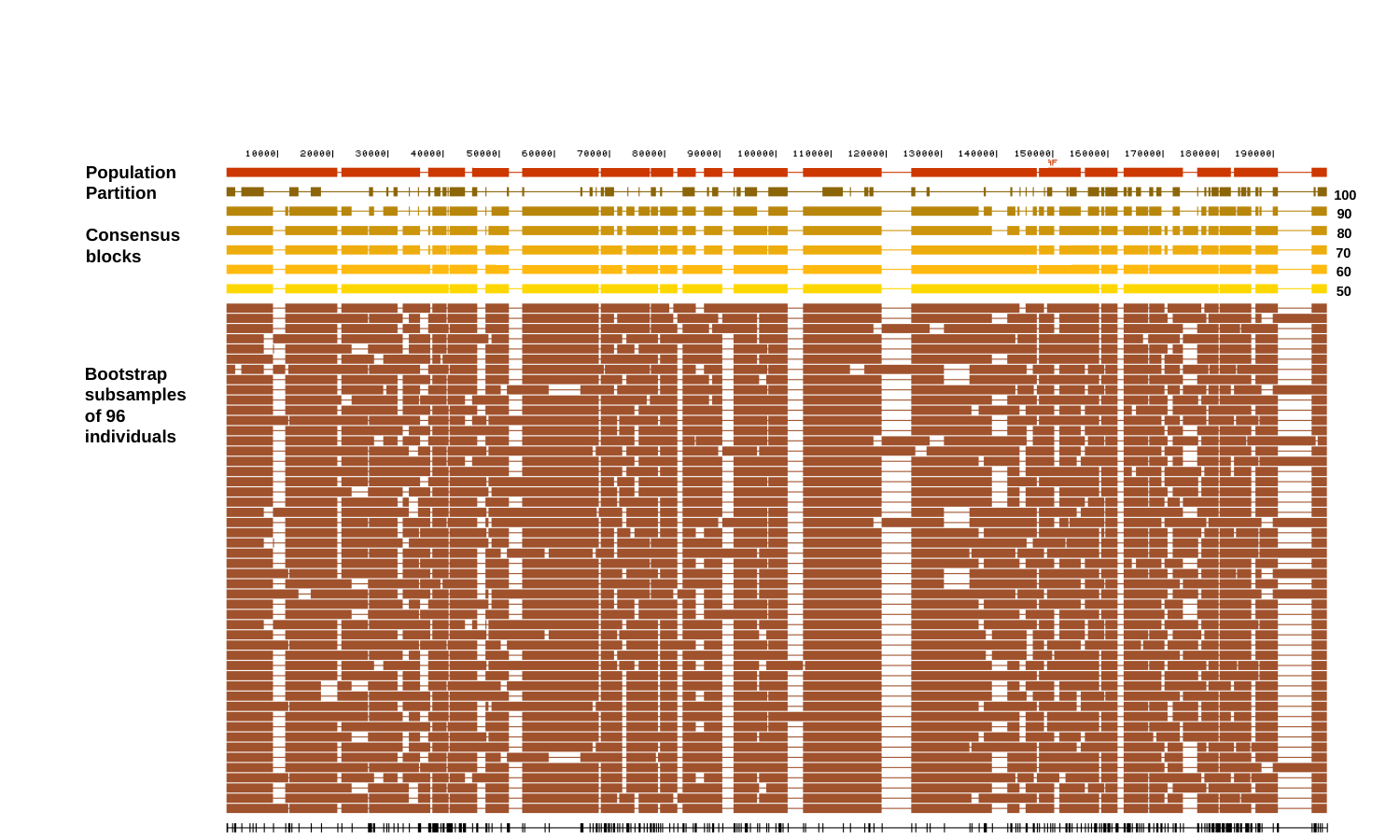

Population Partition
100
90
Consensus blocks
80
70
60
50
Bootstrap subsamples of 96 individuals

Supplement: Additional File 2 — Supplementary Figure 2 shows MDBlocks consensus and bootstrap partitions using all SNPs with at least a 10% MAF for European haplotypes. The first track shows the population partition using all 1000 chromosomes followed by consensus blocks defined at thresholds of 100-50% from bootstrap samples of size 96. The next set of tracks are the first 50 individual bootstrap MDBlocks partitions. [file 1471-2105-6-303-S2.ppt]

## Slide 1
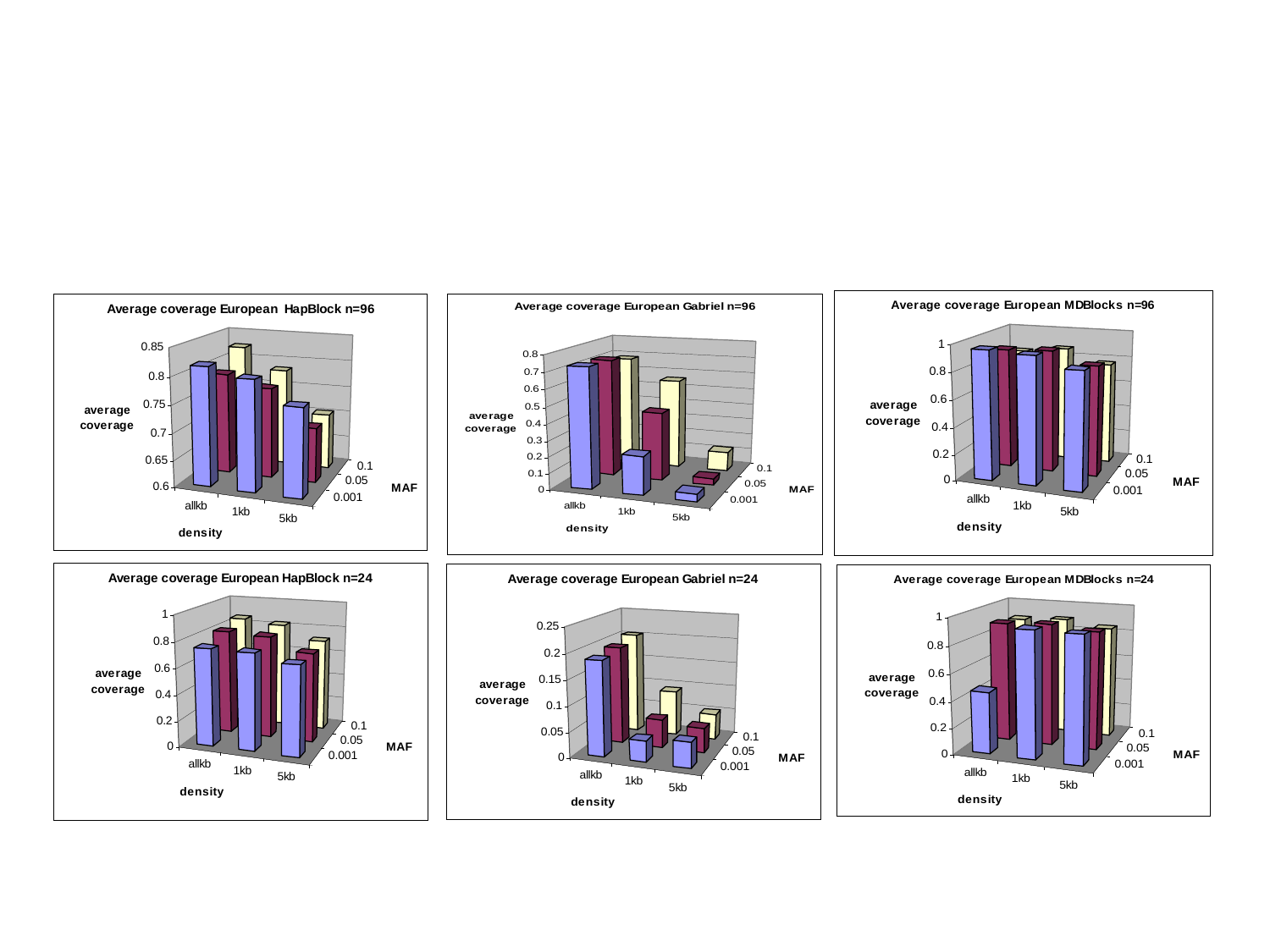

Supplement: Additional File 3 — Supplementary Figure 3 shows 3-d bar plots of the average coverage of HapBlock, Gabriel's method, and MDBlocks partitions on European bootstrap replicates of sizes 96 and 24 at each SNP density and MAF condition. [file 1471-2105-6-303-S3.ppt]

## Slide 1
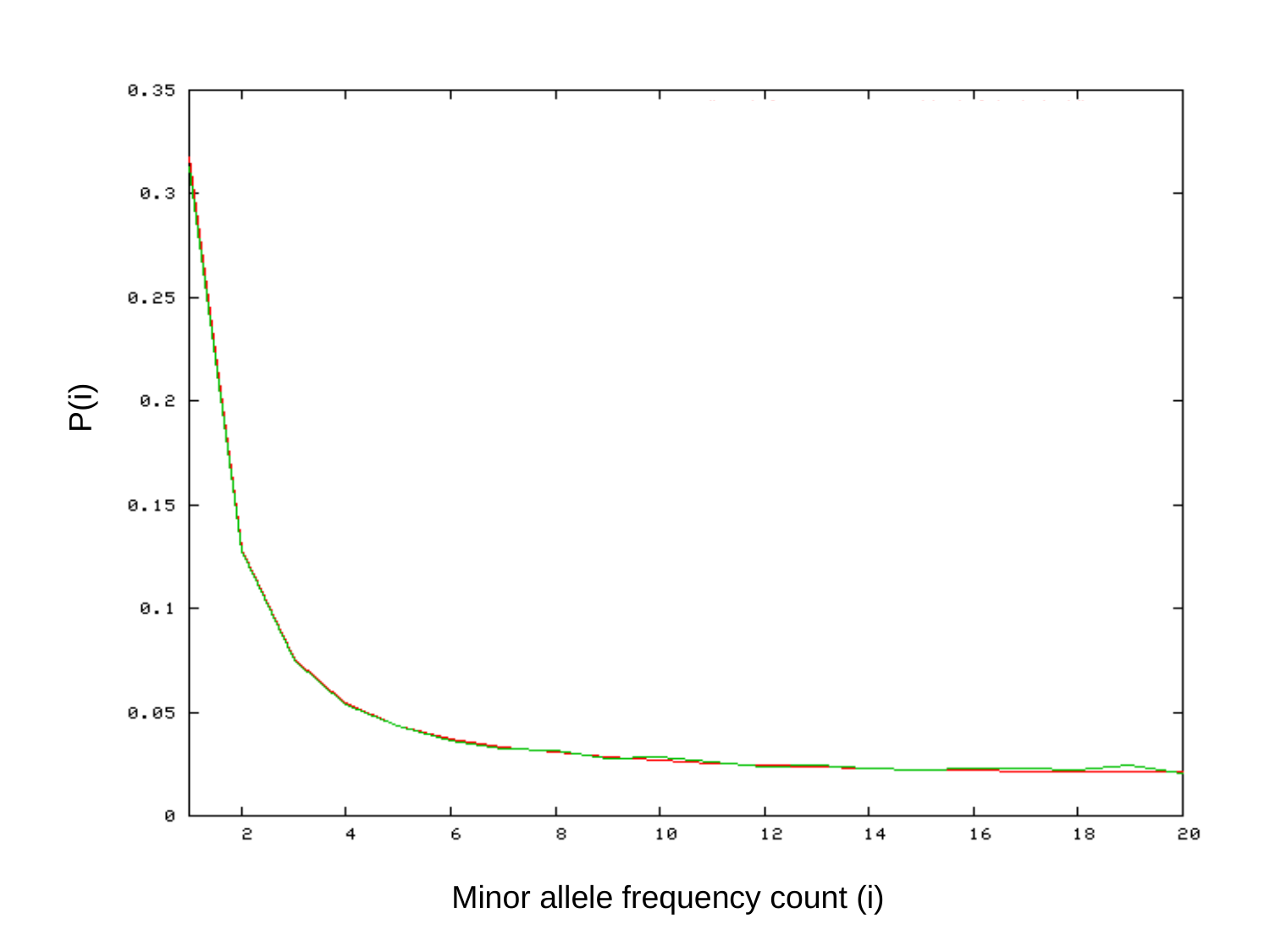

P(i)
Minor allele frequency count (i)

Supplement: Additional File 4 — Supplementary Figure 4 shows the validated European allele frequency spectrum (AFS). The average folded AFS from 200 coalescent genealogies of 41 individuals is plotted in green. The predicted AFS from Marth's mathematical formula is shown in red. [file 1471-2105-6-303-S4.ppt]

## Slide 1
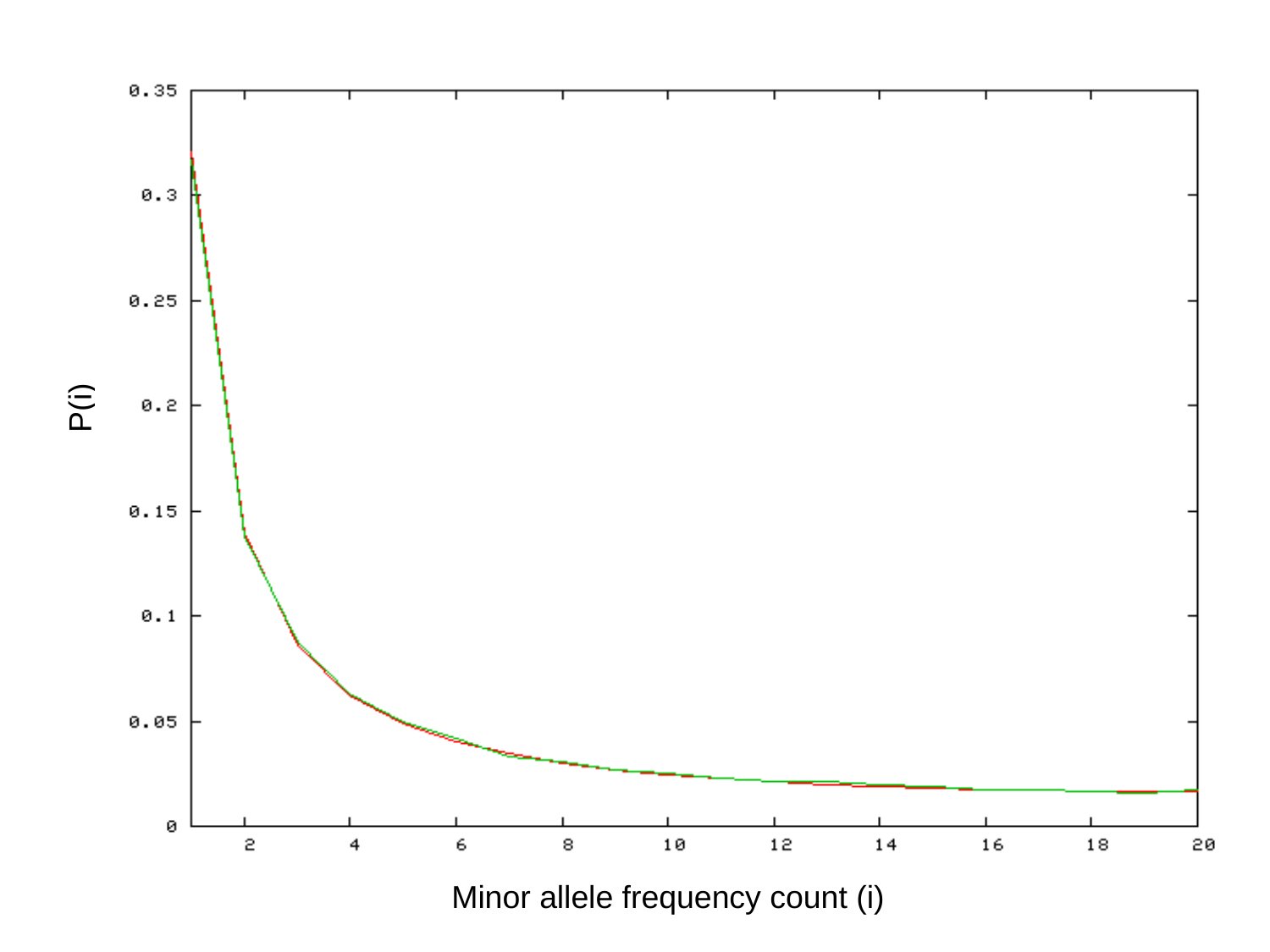

P(i)
Minor allele frequency count (i)

Supplement: Additional File 5 — Supplementary figure 5 shows the validated African American allele frequency specturm (AFS). The average folded AFS from 200 coalescent genealogies of 41 individuals is plotted in green. The predicted AFS from Marth's mathematical formula is shown in red. [file 1471-2105-6-303-S5.ppt]

## Slide 1
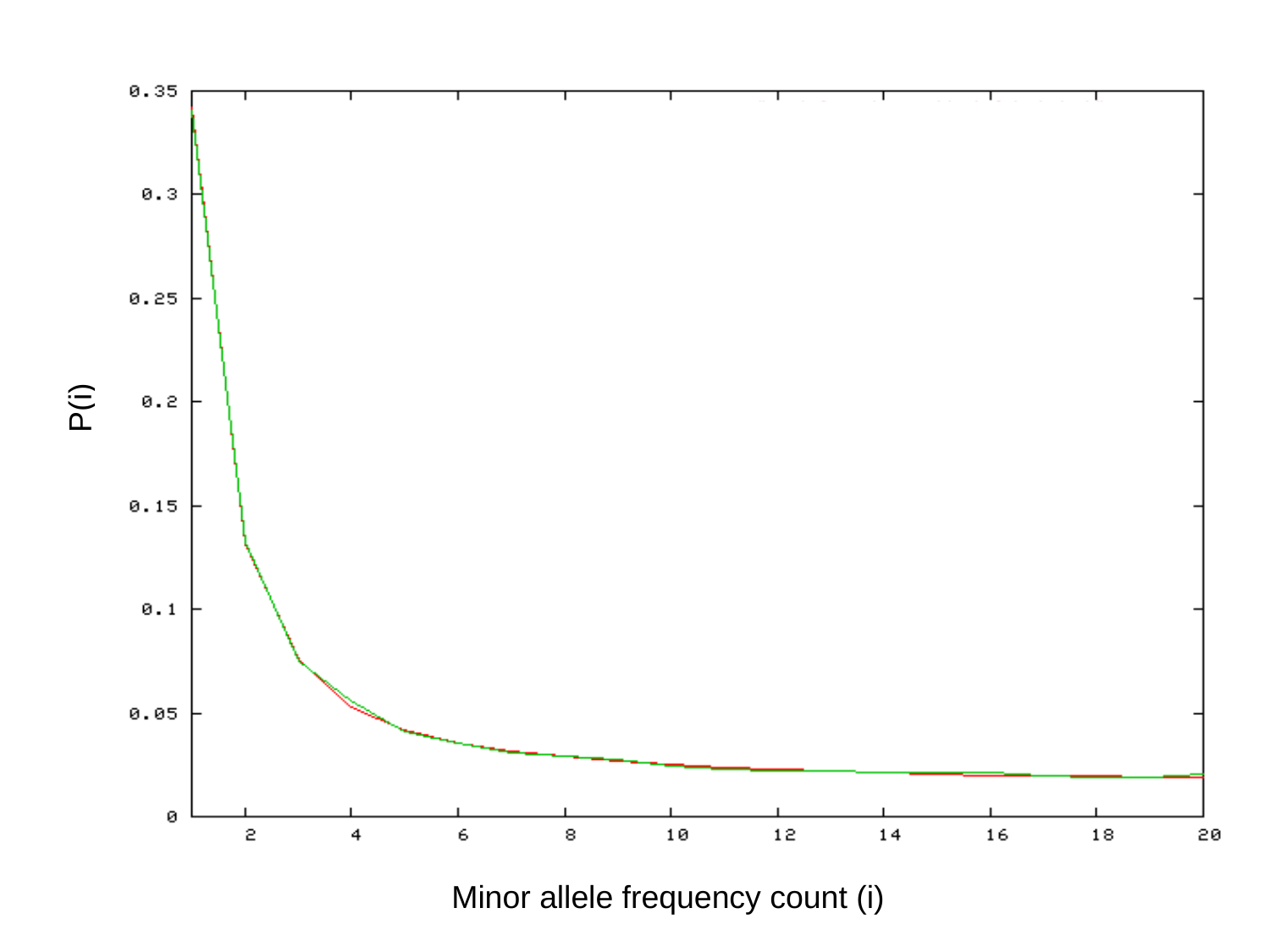

P(i)
Minor allele frequency count (i)

Supplement: Additional File 6 — Supplementary figure 6 shows the validated East Asian allele frequency specturm (AFS). The average folded AFS from 200 coalescent genealogies of 41 individuals is plotted in green. The predicted AFS from Marth's mathematical formula is shown in red. [file 1471-2105-6-303-S6.ppt]
